# Can Serum Levels of Alkaline Phosphatase and Phosphate Predict Cardiovascular Diseases and Total Mortality in Individuals with Preserved Renal Function? A Systemic Review and Meta-Analysis

**DOI:** 10.1371/journal.pone.0102276

**Published:** 2014-07-17

**Authors:** Jing-Wei Li, Cui Xu, Ye Fan, Yong Wang, Ying-Bin Xiao

**Affiliations:** 1 Institute of Cardiovascular Surgery, Xinqiao Hospital, Third Military Medical University, Chongqing, PR China; 2 Medical Department, 305 hospital of PLA, Beijing, PR China; 3 Institute of Respiratory, Xinqiao Hospital, Third Military Medical University, Chongqing, PR China; Mario Negri Institute for Pharmacological Research and Azienda Ospedaliera Ospedali Riuniti di Bergamo, Italy

## Abstract

**Background:**

It is demonstrated that elevated serum levels of alkaline phosphatase (ALP) and phosphate indicate a higher risks of cardiovascular disease (CVD) and total mortality in population with chronic kidney disease (CKD), but it remains unclear whether this association exists in people with normal or preserved renal function.

**Method:**

Clinical trials were searched from Embase and PubMed from inception to 2013 December using the keywords “ALP”, “phosphate”, “CVD”, “mortality” and so on, and finally 24 trials with a total of 147634 patients were included in this study. Dose-response and semi-parametric meta-analyses were performed.

**Results:**

A linear association of serum levels of ALP and phosphate with risks of coronary heart disease (CHD) events, CVD events and deaths was identified. The relative risk(RR)of ALP for CVD deaths was 1.02 (95% confidence interval [CI], 1.01–1.04). The RR of phosphate for CVD deaths and events was 1.05 (95% CI, 1.02–1.09) and 1.04 (95% CI: 1.03–1.06), respectively. A non-linear association of ALP and phosphate with total mortality was identified. Compared with the reference category of ALP and phosphate, the pooled RR of ALP for total mortality was 1.57 (95% CI, 1.27–1.95) for the high ALP group, while the RR of phosphate for total mortality was 1.33 (95% CI, 1.21–1.46) for the high phosphate group. It was observed in subgroup analysis that higher levels of serum ALP and phosphate seemed to indicate a higher mortality rate in diabetic patients and those having previous CVD. The higher total mortality rate was more obvious in the men and Asians with high ALP.

**Conclusion:**

A non-linear relationship exists between serum levels of ALP and phosphate and risk of total mortality. There appears to be a positive association of serum levels of ALP/phosphate with total mortality in people with normal or preserved renal function, while the relationship between ALP and CVD is still ambiguous.

## Introduction

Alkaline phosphatase (ALP) is an enzyme responsible for hydrolyzing phosphate esters and liberating inorganic phosphate. Serum ALP activity is elevated in hepatobiliary and bone diseases such as obstructive jaundice and bone cancer. Phosphorus exists in both organic and inorganic forms in our body. The phosphorus in inorganic form is often combined with calcium within the skeleton, while approximately 15% as a phosphate salt exists in the blood. Circulating ALP and phosphate concentrations often increase in end-stage renal disease, and are associated with higher cardiovascular and total mortality among hemodialysis patients [1], [2]. Previous meta-analyses show that elevated serum levels of phosphorus indicate a higher risk of mortality in population with chronic kidney disease (CKD) [3], [4], with the premise that the relation between the exposure and outcome being linear. However, some researchers have recently found that higher levels of serum ALP and phosphate indicate a higher risk of cardiovascular diseases (CVDs) and total mortality in people without CKD. To our knowledge, no meta-analysis has been conducted yet to study this issue in individuals with normal or preserved renal function, and the shape of the association remains uncertain. Therefore, we conduct a dose-response meta-analysis to quantify the association between serum levels of ALP and phosphate and the risk of CVDs and total mortality in these individuals.

## Methods

Meta-analyses of observational studies were performed following the standard criteria [5].

### Search strategy

PubMed and Embase were searched to identify all relevant articles from inception to 2013 December. Key words used for search included “alkaline phosphatase”, “phosphate”, “hypophosphatemia”, “hyperphosphatemia”, “cardiovascular diseases” and “mortality”. The search strategy was provided in a supplemental file (File S1). Furthermore, we manually searched references of retrieved studies for potentially relevant publications that were not identified in the database search.

### Study selection

Studies were included in this meta-analysis if they meet the following criteria: the exposures were alkaline phosphatase or phosphate; the outcome was risks of CVD (including incidence of CHD events, CVD events and deaths) and total mortality. If one study did not consider stroke as CVD events but reported risks of stroke, then this study was also included in the analysis of CVD events.

Studies were excluded if they lacked HR or RR or numbers of cases and patients for each category (a continuous value per unit was acceptable); or the outcomes were about functional score and calcification. Additionally, reviews, animal studies, letters and conference abstracts without sufficient data were excluded. Whenever more than one article conducted the same study, we combined the results to get a more complete one and avoided the duplication of information.

### Data extraction

The literature search and data extraction were undertaken independently by two authors (J. L. and C. X.) following a standard extraction process. Information on the following parameters was extracted and entered into a database: authors, study name, subjects, publication year, age, gender, country, follow-up years, study types (prospective/retrospective), adjusting variates, diabetes mellitus, patient types (CVD or not), number of cases and events and events and RR (or HR) for all-cause mortality and CVD in each category. The end points were CHD events, CVD deaths, CVD events and total mortality. We firstly manually excluded studies containing patients with only end-stage renal diseases or stages 3–5 CKD or on dialysis through title and abstract screening, but included studies of CKD after full-text reading if the results of participants with eGFR >60 were reported separately (only two were included). We did not exclude studies including CKD patients of general population, but most of these studies took CKD as a parameter for full adjustment. Thus we did our best to exclude those containing participants with eGFR<60. And we collected both partially and fully adjusted RR to compare the results, as it’s reported that adjustments, instead of age, may encumber the comparison between studies [6]. Newcastle-Ottawa quality assessment scale (NOS) [7] was used for quality assessment. When HR or RR was presented only as graph, we used the software g3data (version 1.51, www.frantz.fi/software/g3data.php) to extract the estimates. Any disagreements in the abstracted data were adjudicated by a third reviewer (Y. X).

### Statistical analysis

The number of individuals encountering events, and the partially or fully adjusted HR or RR of ALP (10 UI/L) and phosphate (1 mg/dL) from each study were used. Here the HR was deemed equivalent to RR. Those articles reporting both CVD and total mortality were treated as separate reports. If the continuous RR per unit was unavailable to a study, we used the methods as previous described [in press, doi: 10.1007/s10072-014-1850-1]. In short, we first evaluated a potential linear association between ALP/phosphate levels and risk of outcomes, using restricted three knot cubic spine method (or four knots at percentiles 5%, 35%, 65%, and 95%, if results of the three knot ones show P<0.05). If linearity exists, we used the methods described by Nicola et al [8] to estimate RR of ALP (10 UI/L) and phosphate (1 mg/dL). This method assumes a linear relation between exposure and the logarithm of RR, and estimates the increase in log odds with per unit increase of exposure, taking appropriate account of the non-independence of the RR and transforming these RR into an overall RR as a continuous effect. We assigned the midpoint of the cut points of the class as the dose value, and for open-ended risk factor classes we used the methods described by Nienke et al [6] to get the dose value. The following methods were adopted to determine the event number in categories not reported in a study. If one study reported the total event number and gave RR in the three categories as 1, 1.2, 1.4, we assumed the event number of the third tertile is 1.4 times of that in the reference category, and then calculated the total event number in each category. If the number of events or participants was not given, we preferred to use continuous RR given directly in the study (also in condition which the unit step isn’t 10 UI/L of ALP and 1 mg/dL of phosphate). If P for linearity is less than 0.05, we first got the estimating values directly from the cubic spine estimation, and then used semi-parametric methods described by Frank [9]. In this method, the lowest and the highest groups of ALP or phosphate corresponded to the reference and highest categories, respectively. The middle group corresponded to either the second or the third (or the fourth, if exists) category in the original article, depending on the similarity of the ALP/phosphate in the third or fourth category to the middle group. We performed stratified analyses based on publication year, country, gender, age, diabetes, the NOS score, patient type, number of patients, follow-up duration and types of study in semi-parametric analysis. A similar stratified analysis was conducted in parametric analysis by testing the likelihood ratio and comparing the models derived from the interaction terms or not. We also assessed the publication bias using the Begg’s funnel plots, and the trim-and-fill method was applied to adjust the risk estimates when publication bias existed [10]. We chose a more significant results irrespective of the model used in the trim-and-fill method.

For all analyses, the statistical significance was set at a p value of 0.05, and CIs were calculated at the 95% level. If the results were homogeneous (I^2^<50%, P>0.05), fixed effects models (Inverse-variance method) were utilized, whereas if these results were heterogeneous (I^2^≥50%, P≤0.05), then random effect models (DerSimonian and Laird method) were used. Mantel-Haenszel method was not used because we could not get 2*2 cell counts or the mean and standard deviations but only RR and 95% CI from each study using GLST method. Analyses were performed using Stata software version 11 (StataCorp, College Station, Texas).

## Results

### Study characteristics

**Figure 1:**
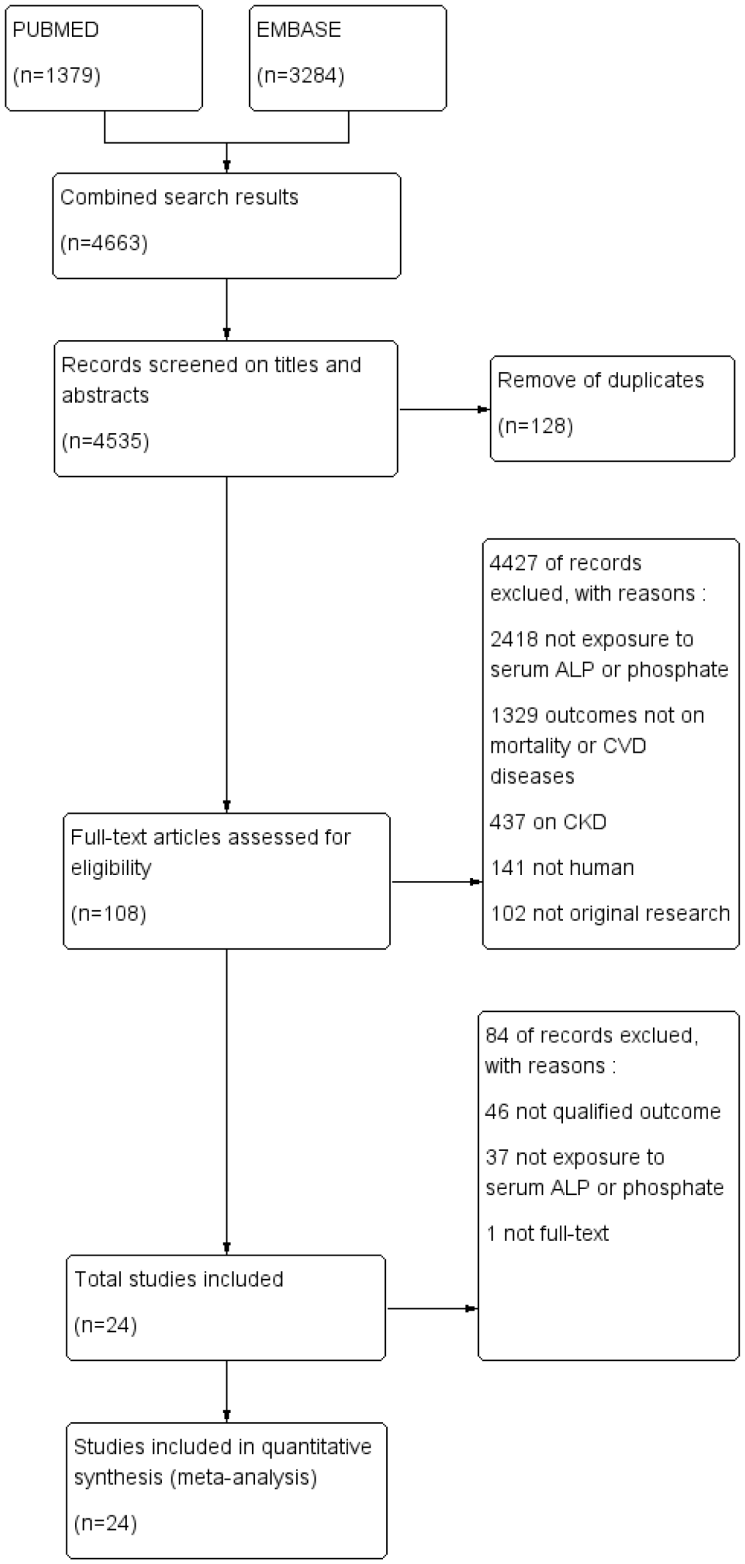
Search strategy and selection process.

We identified 108 studies for further evaluation after reviewing titles, abstracts to exclude the duplicates or irrelevant studies, papers on CKD, case reports and reviews. Then 84 studies were excluded after full-text reading, because of which, 46 studies had no unqualified results, 1 had no full text, and 37 contained patients without exposure to serum ALP or phosphate. Among these excluded ones, Camille P. [11] and Ana Ludimila [12] studied the association between ALP/phosphate and vascular calcification; the research of Jinkwon and his colleagues [13] studied the functional outcomes of cerebrum with ALP and phosphate; Jason P. [14] focused on aortic valve calcification; the study of Gregory [15] was about phosphate and hypertension; and Bernard M.Y [16] investigated the association between alkaline phosphatase and peripheral arterial disease. Finally, a total of 24 trials [16]–[39] with 147634 individuals were identified from 2011 potentially relevant publications (Figure 1). Table 1 summarized the baseline characteristics of included trials. The individuals in studies of Sasiwarang [35] (2013), Edith [26] (2013), Matthew [36] (2010), Ravi D [19] (2007), Robert N. F [23] (2008), Bernard M.Y. C [16] (2009) Tobias E. L [27] (2010), K. M. Fleming [22] (2011), Per-Anton Westerberg [37] (2013), C. J. Bates [38] (2011), Bernard [40] (2009) and Julie R. D [20] (2013) were general population. The studies by Jun-Bean (2013) [29], Jan-P v K [34] (2010), Marcello (2009) [33], Michael [21] (2013), W.-S. [30] (2010) and Grandi [24] (2012) targeted at CVD patients. While CKD patients were the main subjects in Andrew [28] (2013) and Bryan K [41] (2005) studies. We chose patients with CKD stages 1–2 as study subjects in studies like these. The individuals in study of Jun-Bean [18] (2013) all had type II diabetes. Postmenopausal women with osteoporosis and male doctors were components in Yelena S [31] (2011) and Eric N [39] (2011) studies, respectively. The study by Marcello (2005) [33] contained the same subjects as what he chose in 2009 [33]. In his two studies, the former focused on phosphate while the latter was on ALP, which were both included in our analysis. Stephen J. [42] studied the the same objects as those in Robert N. [23] study, but we included the latter because it contained a more complete data. Vidya Raj [43] and Tonelli, M [32] studied the same study population, and we enrolled the latter because it consisted of more suitable subjects. Here we defined preserved renal function as eGFR>60 ml/min/1.73 m^2^. The adjusted information can be seen in Table 1.

**Table 1:** Baseline characteristics of included studies.

Study	Subjects	No.	Duration	Adjusting Factors
Sasiwarang 2013	men without MI or stroke	3381	11	cigarette smoking, alcohol intake, physical activity, social class, age, body mass index, use of antihypertensive drugs, diabetes mellitus, lung function, systolic blood pressure, and estimated glomerular filtration rate, C-reactive protein and von Willebrand factor, CKD(CHD AND CVD events).
Jun-Bean 2013	CAD patients who underwent PCI with DES	1636	2.1	age, gender, diabetes mellitus, hypertension, primary diagnosis as MI at admission, traditional risk factors including smoking status, hypercholesterolemia, use of aspirin, clopidogrel, beta-blockers, angiotensin-converting enzyme inhibitors, statins at the time of index PCI, LVEF, angiographic data including multi-vessel disease, left main disease, ACC/AHA lesion type B2/C, stent length, stent diameter, number of implanted stents and laboratory findings including eGFR, high-sensitivity C reactive protein, hemoglobin, HDL cholesterol, calcium, phosphate, albumin, bilirubin, SGOT, and SGPT
QICKD 2013	people with normal renal function or CKD stages 1–2	Normal: 24184 CKD1∼2∶20356	2.5	known cardiovascular risk factors
Rotterdam Study 2013	inhabitants 55 years or older	5186	19.5	age, sex, education, smoking status, alcohol intake, hypertension, diabetes mellitus, BMI and total cholesterol levels.
Michael Ess 2013	patients with stable heart failure	977	1	sex, age, body mass index, ischemic etiology, left ventricular ejection fraction, systolic blood pressure, New York Heart Association class, estimated glomerular filtration rate, and diuretic and aldosterone antagonist use
NHANES 2005–2006	men and non-pregnant women over the age of 20 years	4155	6.8	age, gender, ethnicity and BMI
W.-S. Ryu 2010	patients after acute stroke	2029	6	age, gender, admission NIH Stroke Scale score, hypertension, diabetes, smoking, heart disease, previous stroke, glucose, serum cholesterol, serum phosphate, glomerular filtration rate, and albumin.
Abramowitz 2010	outpatients with an estimated GFR>60	10743	6.8	age, gender, and race/ethnicity, diagnosis of DM, hypertension, and prior CVD; insurance status; hospitalization within 28 days after the index date; eGFR, corrected serum calcium, serum albumin, hemoglobin, cholesterol, bicarbonate, SGPT, and total bilirubin; and phosphate for the AlkPhos models (top) and AlkPhos for the phosphate models
CARE (2009,2005)	participants with a previous MI	4115	4.9	(2009 ALP) sex, age, race, serum phosphate, alcohol use, fasting serum glucose, use of adrenergic blockers, proteinuria, GFR, systolic blood pressure, angiotensin-converting enzyme inhibitors, serum albumin, red blood cell distribution width, hemoglobin, HDL cholesterol, serum calcium, serum bilirubin, serum glutamic oxaloacetic transaminase, glutamyl transpeptidase and serum glutamate-pyruvate transaminase (2005 Phosphate) sex, age, race, baseline smoking status, diabetic status, waist-to-hip circumference ratio, baseline fasting glucose, baseline aspirin use, baseline GFR, baseline hemoglobin, base line serum albumin, and left ventricular ejection fraction
NHANES III 1988∼1994	civilian noninstitutionalized US population	14716	6	age, sex, race, smoking status, systolic blood pressure, antihypertensive medication use, glomerular filtration rate<60, albuminuria, hemoglobin, red blood cell distribution width, serum albumin, HDL cholesterol, serum calcium, serum phosphorus, serum 25-hydroxyvitamin D, diabetes mellitus, serum bilirubin, C-reactive protein 3 mg/L, alcohol use, serum glutamic oxaloacetic transaminase, and serum glutamate-pyruvate transaminase
BNDNS 2011	community-living 65+ years	1,054	14.5	age,plasma α1-antichymotrypsin
MRC 2011	people aged 75 years and above	13 276	7.2	age, gender, comorbidity group, alcohol intake and WHR group
CHIPS 2005	veterans with chronic renal disease	679	5	age, race, gender, prevalent diabetes, ischemic heart disease, cerebrovascular disease, congestive heart failure, acute renal failure, calcium intake from medications, hemoglobin, serum calcium, the inverse of baseline creatinine, time-averaged creatinine (area under the curve), slope of creatinine, and maximal creatinine concentration during the baseline period.
Framingham Offspring Study 2007	participants in the community free of CVD and CKD	3368	16.1	age, sex, body mass index (calculated as weight in kilograms divided by height in meters squared), diabetes mellitus, systolic blood pressure, treatment for hypertension, smoking, alcohol consumption, total–high-density lipoprotein cholesterol ratio, hemoglobin, serum albumin, estimated glomerular filtration rate, proteinuria, and high-sensitivity C-reactive protein.
ABCD 2009	patients with type 2 diabetes mellitus	950	4.8	Cardiovascular death model: age, sex, race, study drug and treatment arm, systolic and diastolic blood pressure, serum creatinine, serum glucose, serum calcium, non-high density lipoprotein cholesterol and albuminuria status.Cardiovascular event model: age, sex, race, study drug and treatment arm, cardiovascular history, duration of diabetes, systolic blood pressure, serum glucose, serum creatinine and albuminuria status.
MrOS in Sweden 2013	older men in Sweden(aged 69–81 years)	3014	4.5	age
ULSAM 2010	community-based cohort	2176	29.8	age, albumin, eGFR, CG, diabetes, use of antihypertensive medication, systolic and diastolic blood pressures, total cholesterol, triglycerides, body mass index and smoking
KAROLA 2012	population with stable coronary heart disease	1206	8	age, gender, smoking, ventricular function, history of myocardial infarction, history of hypertension, diabetes, creatinine clearance, treatment with β-blockers, calcium antagonists, lipid lowering drugs, angiotensin converting enzyme inhibitors, aspirin and diuretics, alcohol consumption during the previous 12 months, hemoglobin, body mass index, triglycerides, total cholesterol, low density lipoprotein cholesterol, high density lipoprotein cholesterol, fasting glucose and albumin (only in models for uncorrected calcium)
MrOS in USA 2013	community-dwelling men 65 years or older	1,325	9.3	age and race, estimated glomerular filtration rate and micro albuminuria, prevalent cardiovascular disease, diabetes, systolic blood pressure, blood pressure medication use, tobacco use (current, former, never), body mass index, total cholesterol level, high-density lipoprotein cholesterol level, and lipid medication use
Jan-Peter 2010	patients scheduled for major vascular surgery	1,798	3.6	age, gender, ischemic heart disease, cerebrovascular disease, current smoking, baseline eGFR, dyslipidemia, diabetes mellitus, heart failure, COPD, systolic blood pressure and body mass index.
MORE 2011	postmenopausal women with osteoporosis	7705	4	race, age, albumin, smoking status, alcohol consumption, BMI, systolic blood pressure, diastolic blood pressure, prior history of CVD, history of diabetes, diuretic use, aspirin use, lipid lowering medication use at baseline, HDL, hemoglobin, LDL, triglycerides, eGFR, 25(OH)D, treatment assignment, calcium, PTH.
ARIC 2008	community-dwelling adults	15732	12.6	Age, comorbid conditions, demographic characteristics, albumin, and estimated glomerular filtration rate
HPFS 2011	male doctors	1259	10	age, month and year of blood collection, and smoking status) and also for family history of MI before the age of 60 years, alcohol intake, physical activity, BMI, race, multivitamin use, region, marine ω-3 intake, history of diabetes mellitus, history of hypertension, and fasting status, FGF23, PTH, phosphorus, 25(OH)D, HDL cholesterol, LDL cholesterol, triglycerides, uric acid, and creatinine
Doron 2013	patients with acutemyocardial infarction	1663	3.75	gender, age, eGFR, hemoglobin, previous infarction, diabetes mellitus, hypertension, smoking, baseline hemoglobin, serum calcium, ST elevation infarction, Killip class, LVEF, coronary revascularization

### Association between serum levels of ALP and phosphate and risk of CVD

**Figure 2:**
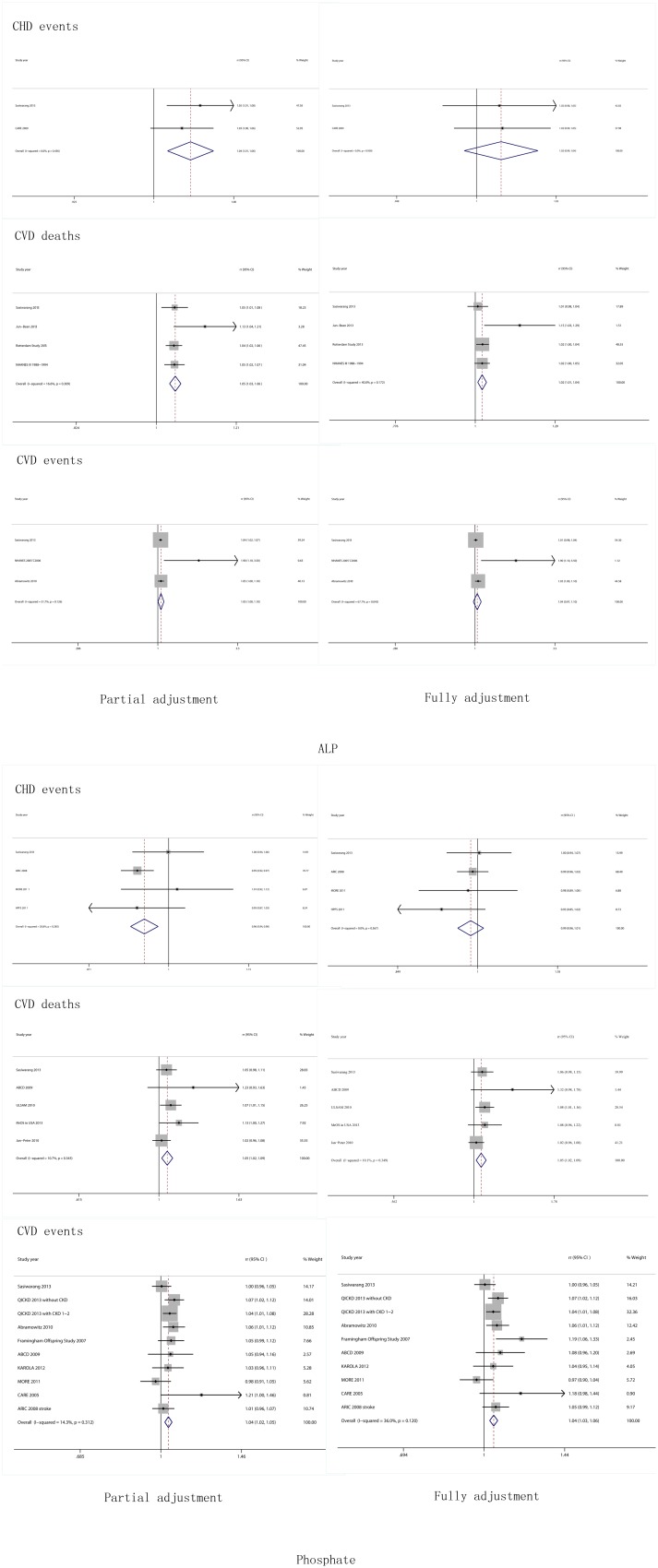
Forest plots for risks of cardiovascular diseases associated with levels of serum ALP and phosphorus.

As shown in Figure 2, the RR of ALP (10 UI/L)for CHD events was 1.04 (95% CI: 1.01–1.06, P = 0.436 for heterogeneity, I^2^ = 0.0%, partial adjustment); for CVD deaths and events was 1.02 (95% CI: 1.01–1.04, P = 0.172 for heterogeneity, I^2^ = 40%) and 1.05 (95% CI: 1.00–1.10, P = 0.126 for heterogeneity, I^2^ = 51.7%, partial adjustment), respectively; the RR of phosphate (1 mg/dL) for CHD events was 0.99 (95% CI: 0.96–1.01, P = 0.567 for heterogeneity, I^2^ = 0%); for CVD deaths and events was 1.05 (95% CI: 1.02–1.09, P = 0.349 for heterogeneity, I^2^ = 10.1%) and 1.04 (95% CI: 1.03–1.06, P = 0.120 for heterogeneity, I^2^ = 36.0%), respectively. For results of the linear relationship between ALP/Phosphate and CVD, please refer to Supplementary Material (File S2).

### Association between serum levels of ALP and phosphate and risk of total mortality

**Figure 3:**
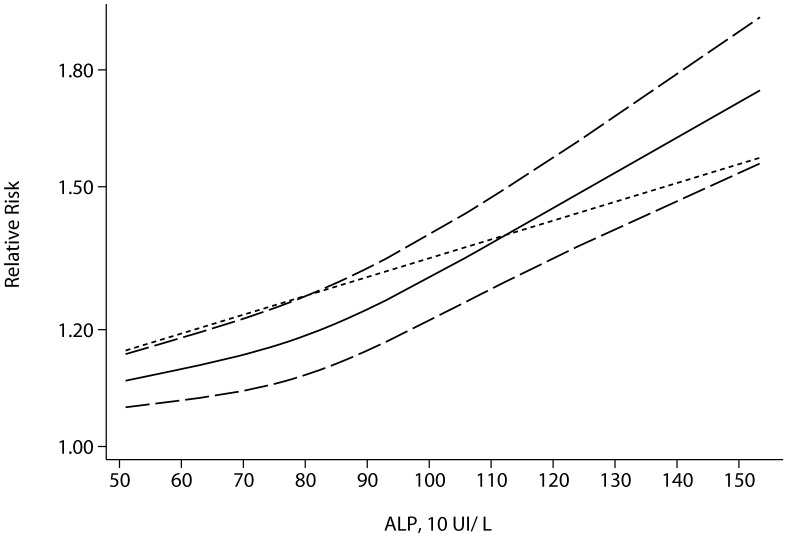
Non-linear relationships of ALP with risk of total mortality. Short-dashed line represents linear regression. Other line represents cubic spline regression.

**Figure 4:**
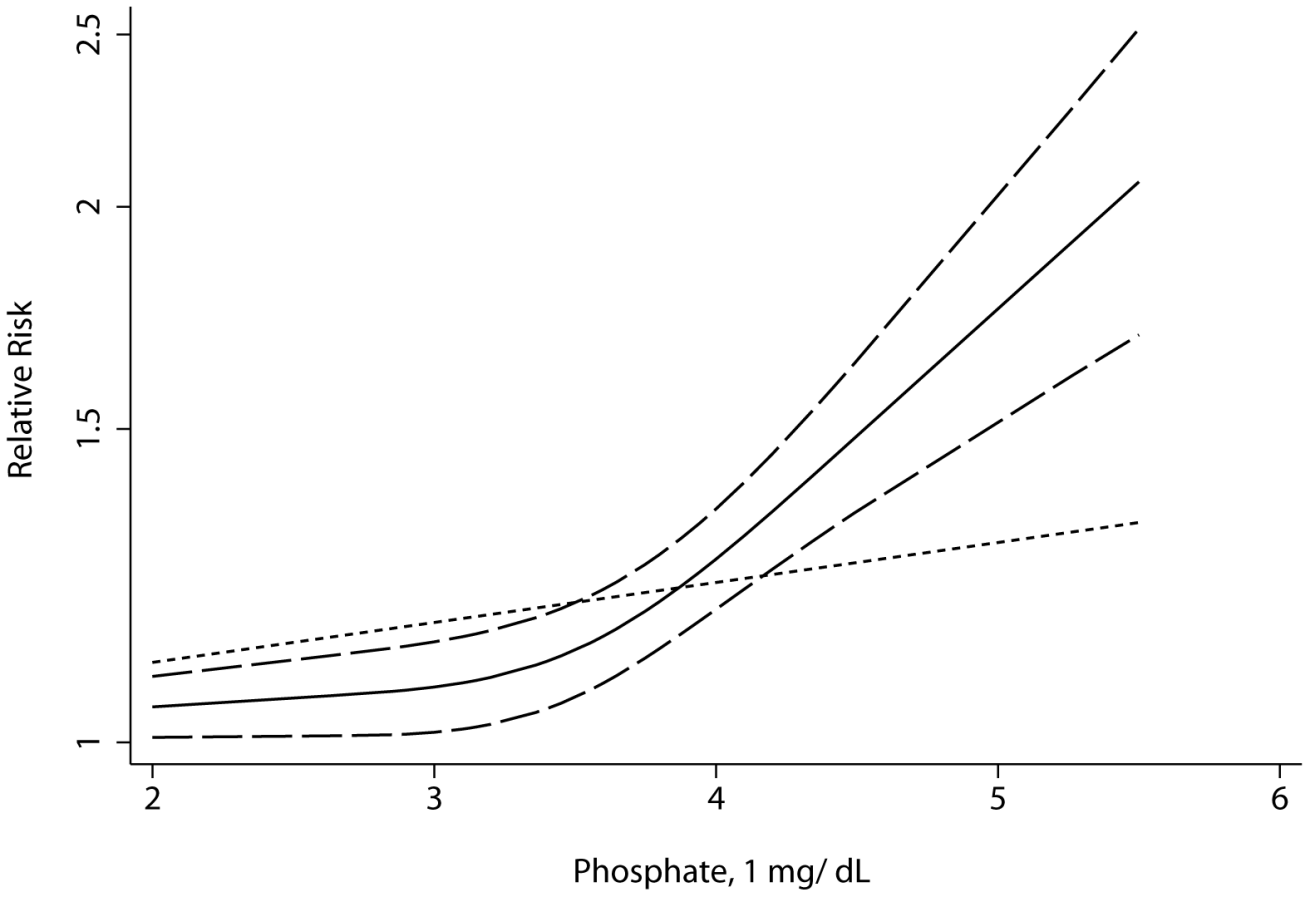
Non-linear relationships of phosphate with risk of total mortality. Short-dashed line represents linear regression. Other line represents cubic spline regression.

We observed a non-linear association between serum levels of ALP/phosphate and risk of total mortality (P for non-linearity = 0.0002 and <0.0001, respectively) under full adjustment (Figure 3, 4). Similar trends were observed under partial adjustment (data not shown). The results reported here were under full adjustment. Compared with the reference ALP (51 UI/L), the RR directly from the cubic spline model for total mortality was 1.07 (95% CI, 1.05–1.09) for ALP = 79 UI/L, and 1.34 (95% CI, 1.26–1.42) for ALP = 124 UI/L, with substantial heterogeneity (Pr = 0.0001). Compared with the reference (Phosphate 2 mg/dL), the RR directly from the cubic spline model for total mortality was was 1.07 (95% CI, 1.04–1.09) for phosphate = 3.45 mg/dL, and 1.42 (95% CI, 1.29–1.56) for phosphate = 4.5 mg/dL, with little heterogeneity (Pr = 0.71). We also used semi-parametric methods to estimate the RR for ALP and phosphate. Compared with the reference category of ALP (<70 UI/L), the pooled RR for total mortality was 1.14 (95% CI, 1.09–1.20, P = 0.057, I^2^ = 48.9%) for the median ALP group (70–90 UI/L), and 1.57 (95% CI, 1.27–1.95, P<0.001, I^2^ = 90.1%) for the high ALP group (>90 UI/L). The RR of phosphate for total mortality was 1.08 (95% CI, 1.03–1.13, P = 0.38, I^2^ = 6.6%) for the median phosphate group (3–4 mg/dL), and 1.33 (95% CI, 1.21–1.46, P = 0.003, I^2^ = 57.5%) for the high phosphate group (>4 mg/dL) (Figure 4). We found an inflexion point at phosphate = 3.5 mg/dL.

### Subgroup analyses

Subgroup analyses were conducted to determine the total mortality related to ALP and phosphate (Table 2, 3). The subgroup analyses were defined according to study type, study location, number of participants, duration of follow-up, publish publication year, gender, age, diabetes mellitus, the NOS score and patient types (CVD or not). The subgroup analysis of study type was unprocurable to ALP because only one study included was retrospective. A high level of ALP predicted a higher incidence of total mortality in the Asians than that in the Europeans and Americans (RR: 3.1, 95% CI: 2.2–4.3 for the Asians; RR: 1.3, 95% CI: 0.97–1.7 for the Europeans and RR: 1.4, 95% CI: 1.2–1.7 for the Americans; P<0.001); in male than that in female(RR: 2.0, 95% CI: 1.2–3.1 for male and RR: 1.4, 95% CI: 1.4–1.8 for female; P<0.001);in shorter follow-up than that in longer follow-up (RR: 2.0, 95% CI: 1.3–3.1 for duration<6 years and RR: 1.4, 95% CI: 1.1–1.8 for duration>6 years; P<0.001); in patients with serious diabetes than that in patients with mild diabetes (RR: 2.0, 95% CI: 1.2–3.1 for diabetes >11% and RR: 1.4, 95% CI: 1.1–1.5 for diabetes <11%; P<0.001); in CVD patients than that in non-CVD patients (RR: 2.4, 95% CI: 1.3–4.5 for CVD patients and RR: 1.4, 95% CI: 1.1–1.7 for non-CVD ones; P<0.001), in study with lower NOS score than that in study with higher NOS score (RR: 2.1, 95% CI: 1.4–3.0 for NOS<7 and RR: 1.3, 95% CI: 1.0–1.6 for NOS> = 7; P<0.001). A high level of phosphate also predicted a higher incidence of total mortality in patients with CVD (RR: 1.5, 95% CI: 1.3–1.8 for CVD patients and RR: 1.3, 95% CI: 1.2–1.4 for non-CVD ones; P<0.003), in patients with serious diabetes than that in patients with mild diabetes (RR: 1.5, 95% CI: 1.3–1.6 for diabetes >11% and RR: 1.2, 95% CI: 1.1–1.3 for diabetes <11%; P<0.001). Similar analyses were conducted using parametric methods. Publication year, gender, number of patients, duration of follow-up and diabetes mellitus had the same trends for ALP while others did not. No significant difference was found in any of the subgroups for phosphate.

**Table 2:** Stratified analyses of relative risk of total mortality for ALP high group.

Item	Subgroup	No	RR	95% CI		P. Het	I^2^	P. test
country	Europe	3	1.301	0.970	1.743	0.000	92.4	0.079
	Asia	2	3.072	2.206	4.277	0.341	0.0	<0.001
	America	3	1.438	1.215	1.701	0.155	46.3	<0.001
Publish year	>2010	4	1.500	1.087	2.070	0.000	92.2	0.014
	<2010	4	1.651	1.255	2.172	0.001	80.5	<0.001
Male	>60%	4	1.967	1.237	3.129	0.000	86.7	0.004
	<60%	4	1.388	1.080	1.785	0.000	92.2	0.010
No. pat	>4000	4	1.776	1.151	2.740	<0.001	92.2	0.009
	<4000	4	1.510	1.308	1.743	0.094	53.0	<0.001
Age	>64	4	1.530	1.099	2.129	<0.001	93.8	0.002
	<64	4	1.603	1.226	2.097	0.008	74.9	0.012
Duration (year)	>6	4	1.377	1.062	1.786	0.000	92.1	0.016
	<6	4	1.974	1.267	3.077	0.000	86.5	0.003
Diabetes mellitus	>11%	4	1.967	1.237	3.129	0.000	86.7	0.004
	<11%	4	1.388	1.080	1.785	0.000	92.2	0.010
Patient type	Non-CVD	5	1.354	1.102	1.663	0.000	89.6	0.004
	CVD	3	2.422	1.311	4.475	0.002	84.5	0.005
NOS score	> = 7	4	1.291	1.040	1.603	0.000	89.0	0.020
	<7	4	2.081	1.445	2.996	0.003	79.0	0.000

No = number of included study; P. Het = P for heterogeneity; P. test = P for test;

**Table 3:** Stratified analyses of relative risk of total mortality for phosphate high group.

Item	Subgroup	No	RR	95% CI		P. Het	I^2^	P. test
country	Europe	8	1.357	1.171	1.572	0.001	70.9	<0.001
	America	5	1.313	1.177	1.466	0.300	18.1	<0.001
Publish year	> = 2011	9	1.448	1.199	1.748	0.000	71.6	<0.001
	<2011	6	1.255	1.161	1.357	0.497	0.0	<0.001
Male	>75%	7	1.303	1.166	1.455	0.374	7.1	<0.001
	<75%	7	1.360	1.165	1.587	0.000	75.6	<0.001
No. pat	>3000	7	1.346	1.228	1.475	0.752	0.0	<0.001
	<3000	8	1.328	1.143	1.542	0.001	71.2	<0.001
Age	>62	8	1.345	1.154	1.567	0.013	60.8	<0.001
	<62	6	1.367	1.188	1.573	0.053	54.2	<0.001
Duration (year)	>7	7	1.292	1.144	1.460	0.009	65.0	<0.001
	<7	7	1.374	1.184	1.595	0.106	40.9	<0.001
Study type	Prospective	10	1.291	1.158	1.439	0.025	52.8	<0.001
	Retrospective	5	1.397	1.154	1.692	0.014	58.1	<0.001
Patient type	Non-CVD	9	1.286	1.158	1.429	0.344	10.9	<0.001
	CVD	4	1.530	1.297	1.806	0.927	0.0	<0.001
Diabetes mellitus	>11%	7	1.465	1.317	1.630	0.380	6.2	<0.001
	<11%	6	1.167	1.077	1.265	0.296	18.1	<0.001
NOS score	> = 7	6	1.263	1.118	1.427	0.017	63.7	<0.001
	<7	9	1.391	1.217	1.589	0.123	37.0	<0.001

No = number of included study; P. Het = P for heterogeneity; P. test = P for test;

### Publication bias

Publication bias for associations between high levels of ALP/phosphate and total mortality was assessed by Begg’s tests (p = 0.007 and p = 0.010, respectively), which was confirmed using parametric methods (data not shown). The RR estimates altered (RR = 1.11, 95% CI: 1.07–1.17, 5 studies added for high ALP group and RR = 1.21, 95% CI: 1.09–1.33, 6 studies added for high phosphate group) after using the trim-and-fill method to adjust the potential publication bias.

## Discussion

This is the first meta-analysis, to our knowledge, to assess the association of serum levels of ALP and phosphate with the risk of death and cardiovascular diseases in individuals without CKD 3∼5. Our research, which includes 24 clinical trials with 147634 patients, identifies a non-linear association between serum ALP/phosphate level and the risk of total mortality. Elevated serum levels of ALP and phosphate indicate a higher total mortality in people with normal or preserved renal function. The relationship between ALP and cardiovascular diseases is still ambiguous.

To date, the majority of studies about the relation of ALP and phosphate levels with total mortality have found a significant association between serum ALP level and total mortality in individuals without CKD3∼5. For example, Jun-Bean et al. [29] analyzed data from 1636 Korean coronary artery disease (CAD) patients after receiving percutaneous coronary intervention (PCI) with almost 2 years of follow-up, finding that the risk of total mortality increases in patients with a higher level of ALP. This study was carried out among Asians with CAD, and 37.3% of included subjects were diabetic patients. Our subgroup analysis shows that Asian ethnic origin, CVD and diabetes affect the prediction of ALP on total mortality, which may explain why this study obtains the most significant result. Yelena et al. [31] studied postmenopausal women with osteoporosis and found that phosphorus level was not associated with a higher risk of total mortality during 4 years of follow-up. Only a small group of subjects had diabetes in this study (<4%), and the subjects all suffered from osteoporosis. Osteoporosis alone is associated with fibroblast growth factor (FGF)-23, which may influence the level of phosphate [44]. These factors may explain why this study does not get significant result.

Our study shows that ALP is positively associated with CVD deaths irrespective of adjustment methods, while elevated ALP reflects a higher incidence of CHD and CVD events only after partial adjustment. The association between ALP and CVD deaths is weakened but still exists after full adjustment, which implies that higher levels of ALP play such roles other than adjustment factors, such as GFR, diabetes mellitus, CRP and hepatic dysfunction. On the other hand, the association between ALP and CHD and CVD events disappears after full adjustment. GFR, diabetes mellitus, hypertension and CRP are full adjustment factors, which may influence the effect of ALP on CHD and CVD events. We speculate that the true association between ALP and CHD/CVD events is likely to be modest; the difference in ALP measurements and covariate adjustment factors such as diabetes mellitus may result in changes in the strength and even the significance of the associations, thus leading to different conclusions. Higher serum levels of ALP have been associated with inflammation [35] and calcification [29]. ALP level is positively associated with CRP [40] and neutrophil to lymphocyte ratio (N/L) [45], and is elevated in vessels with medial calcification [46]. Thus inflammation and calcification could play a role in the association of ALP with CVD deaths.

We found that higher serum levels of phosphate increase the incidence of CVD events and deaths, irrespective of the adjusting method, but not that of CHD events. We compare our results with previous results regarding CKD [3], finding that although the predicting trend of CVD events is the same, the strength of association of phosphate with CVD events in non-CKD is weaker than that of CKD. Our previous work has found serum phosphate is not associated with risk of stroke, here we find serum phosphate is associated with CVD events, which imply that serum phosphate may be only associated with some kinds of CVD events such as coronary artery disease and heart failure but not stroke [in press, doi: 10.1007/s10072-014-1850-1]. Serum phosphate levels are significantly associated with age, female gender, diabetes mellitus, hypertension and hypercholesterolemia, but not with eGFR [47]. This is partially confirmed in another research, which shows that the relationship between serum phosphorus level and renal function is biphasic, with no association between phosphorus and renal function when eGFR is >60 and a linear relationship when eGFR is <60. [48] These previous results indicate that serum phosphate level may be an independent predictor of cardiovascular events and deaths in individuals without CKD 3∼5, which is confirmed in our meta-analysis. The serum level of phosphate is regulated by a balance between dietary intake, absorption from the gastrointestinal tract, storage in the skeleton, and urinary excretion [21]. High serum phosphate level is associated with more favorable profiles of cardiovascular risk factors [49] and calcification [50]. Indeed elevated phosphate level may directly stimulate smooth muscle cells to change phenotypically into osteoblast-like cells [20]. High serum level of phosphate can also cause degradation of the extracellular matrix, change of osteochondroge [51] and increase the production of reactive oxygen species [52] and stimulate an osteoblastic transcriptional program in the vasculature [53] in vascular smooth muscle. Serum phosphate level is a novel pre-clinical biomarker for cardiovascular calcification [54]. Thus calcification potentially establishes the association of ALP level with CVD deaths and events.

**Figure 5:**
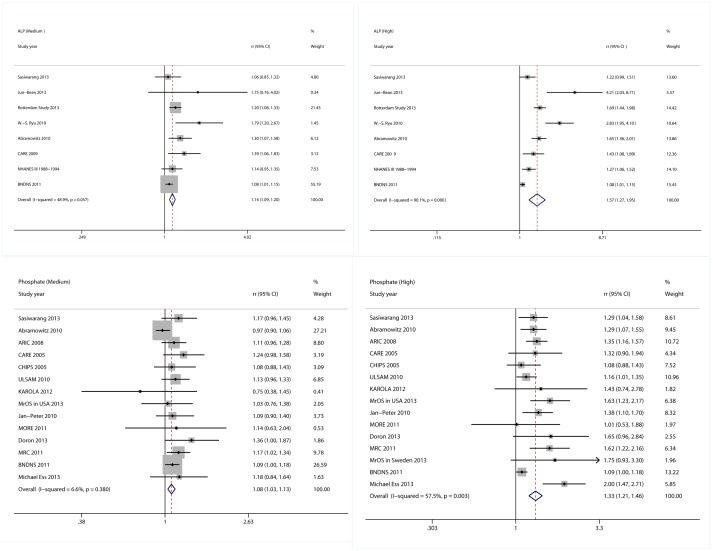
Semi-parametric meta-analysis of median and high levels of ALP and phosphate compared with reference levels.

High serum levels of ALP and phosphate are associated with an increasing incidence of total mortality. We found a non-linear association of ALP and phosphate levels with total mortality, and this association exists even in the normal range (the medium group, Fig. 5), but the risk rises dramatically at a high level (Fig. 3, 4). The association may be partly caused by hypovitaminosis D, which is associated with elevated serum ALP and phosphate levels. Hypovitaminosis D is associated with greater plasma renin activity, inflammation, and higher blood pressure [21], and FGF-23 may play such roles in similar indirect ways, both of which are called phosphate-responsive hormones [55]. We found a turning point of serum phosphate level at about 3.5 mg/dL, beyond which the incidence of total mortality elevated enormously. People with a high serum phosphate level may need special care. We can not explain why some healthy people have higher serum levels of phosphate than the others, and genetic variants account for little of phosphate elevation [55], which needs to be explained by future study. Heterogeneity exists in both high ALP and phosphate group analyses, which we attribute to differences among studies in sample sizes, characteristics of the populations, measurement standards for ALP and phosphate, statistical adjustments and different grouping methods.

We found that ALP was not robust in subgroup analysis. Indeed serum ALP level is elevated in various conditions including liver and bone diseases, various kinds of cancers, chlorpropamide therapy, infectious mononucleosis, leukemoid response to infection, hormonal contraception, pregnancy, and hyperthyroidism. Different study populations may contribute to the diversity. Our research has found that elevated ALP level is more effective in predicting the risk of total mortality in Asian people and in male gender. It is reported that ALP activity is associated with male gender but not with age in adult group. [56] Both ALP and phosphate levels are more effective in predicting the risk of total mortality of people with diabetes and cardiovascular diseases. It is reported that 38% of diabetic patients have elevated serum alkaline phosphatase levels, mean fasting serum glucose level is positively associated with ALP, and bone ALP is the predominant type, which may be caused by diabetic bone diseases [57]. Indeed 9 out of 15 studies on the association of phosphate level with total mortality have shown an ascending incidence of diabetes in higher categories of phosphate (data now shown). Elevated ALP level is also observed in AMI patients [58]. We find that CVD and diabetes are the two main factors influencing heterogeneity of the association between phosphate level and total mortality, as heterogeneity in subgroup analysis by these two factors decreases apparently. The subgroup analysis of ALP by the NOS score shows different estimates between the groups. Patients with CVD and diabetes are more common in the NOS<7 group, which can partly explain the phenomenon.

Our findings raise the possibility that lowering serum ALP and phosphorus levels may decrease CVD or total mortality rates in the general population. Prior studies show that oral phosphorus binders fail to lower serum phosphorus concentration. A better choice to reduce it may be to limit the dietary phosphorus intake [59], which needs to be confirmed by future clinical trials.

It should be noted that severe publication bias exists in both analyses of ALP and phosphate. We have used the trim-and-fill method to adjust the risk estimates. We speculate that the publication bias may partly originate from heterogeneity among tests as we find that publication bias is mild in medium-level phosphate group (P for Begg’s test = 0.238), while it is serious in high-level phosphate group (P for Begg’s test = 0.008). The demarcation among reference, median and high categories differ dramatically among different studies, which may explain this phenomenon. Previous researchers have found that publication bias can be attributed to other factors such as heterogeneity. [60].

This meta-analysis has several limitations. Firstly, some studies do not provide the number of cases or participants in each category. Although we have used the continuous RR or estimated them as described in Methods, misestimation of RR is still inevitable. Secondly, mild to severe publication bias exists. We have tried various methods in subgroup analysis to explain why it exists; We carefully observed the funnel plots and suspected that small studies showing no beneficial effects were missing or heterogeneity among studies may partly explain the bias, since standard error of log RR may not reflect number of participants in each study. The trim-and-fill method is used to amend the results. Thirdly, factors of full adjustment vary among studies included. The author of an earlier meta-analysis of phosphate with the same research aim considered 5 adjusting covariates more suitable [3], which is similar to our partial adjustment group. And another research on methodology indicates that adjustment may hamper the estimation [6]. We find that adjustment can influence the results especially regarding the association between ALP and CVD. Both adjusted and un-adjusted results are presented if such phenomenon exists. Last, we does not conduct the analysis based on kidney function (subgroups: normal kidney function and CKD 1∼2), because our subjects mainly consist of community-based cohorts and some particular patients (e.g. population with stable CHD) without separately analyzing CKD patients. The number of studies on CKD 1∼2 is not enough to make a subgroup analysis. However, renal function serves as adjusting variants in most included studies, and has been adjusted in results of fully adjusted groups.

## Supporting Information

Checklist S1 **Prisma checklist.**
(DOC)

File S1 **Search Strategy.**
(DOCX)

File S2 **Results of the linear relationship between ALP/Phosphate and CVD.**
(DOCX)
