# Supplementary material for: Can Serum Levels of Alkaline Phosphatase and Phosphate Predict Cardiovascular Diseases and Total Mortality in Individuals with Preserved Renal Function? A Systemic Review and Meta-Analysis
Source: PLoS One. 2014 Jul 17;9(7):e102276. doi: 10.1371/journal.pone.0102276 (PMC4102523; doi:10.1371/journal.pone.0102276)
Supplement: File S1 — Search Strategy. (DOCX) [file pone.0102276.s002.docx]

For PubMed:

| #1 | "Phosphates/blood"[Mesh] |
| --- | --- |
| #2 | "Phosphorus, Dietary/blood"[Mesh] |
| #3 | "Hypophosphatemia"[Mesh] |
| #4 | "Hyperphosphatemia"[Mesh] |
| #5 | "Alkaline Phosphatase/blood"[Mesh] |
| #6 | #1 OR #2 OR #3 OR #4 OR #5 |
| #7 | "Mortality"[Mesh] |
| #8 | "Cardiovascular Diseases"[Mesh] |
| #9 | #7 OR #8 |
| #10 | #6 AND #9 |

For Embase:

| #1 | 'phosphate blood level'/exp |
| --- | --- |
| #2 | 'alkaline phosphatase blood level'/exp |
| #3 | hypophosphatemia'/exp |
| #4 | 'hyperphosphatemia'/exp |
| #5 | 'mortality'/exp |
| #6 | 'cardiovascular disease'/exp |
| #7 | #1 OR #2 OR #3 OR #4 |
| #8 | #5 OR #6 |
| #9 | #7 AND #8 |
| #10 | #9 AND ([article]/lim OR [article in press]/lim OR [letter]/lim) AND ([adolescent]/lim OR [young adult]/lim OR [adult]/lim OR [middle aged]/lim OR [aged]/lim OR [very elderly]/lim) AND [humans]/lim |
