# Supplementary material for: Can Serum Levels of Alkaline Phosphatase and Phosphate Predict Cardiovascular Diseases and Total Mortality in Individuals with Preserved Renal Function? A Systemic Review and Meta-Analysis
Source: PLoS One. 2014 Jul 17;9(7):e102276. doi: 10.1371/journal.pone.0102276 (PMC4102523; doi:10.1371/journal.pone.0102276)
Supplement: File S2 — Results of the linear relationship between ALP/Phosphate and CVD. (DOCX) [file pone.0102276.s003.docx]

**Linear relationship**

CHD-EVENTS

| A | B | P for Nonlinearity |
| --- | --- | --- |
| ALP | Partial adjustment | 0.8790 |
|  | Fully adjustment | 0.5409 |
| phosphate | Partial adjustment | 0.6339 |
|  | Fully adjustment | 0.1110 |

CVD-DEATHS

| A | B | P for Nonlinearity |
| --- | --- | --- |
| ALP | Partial adjustment | 0.05* |
|  | Fully adjustment | 0.9232 |
| phosphate | Partial adjustment | 0.1398 |
|  | Fully adjustment | 0.2573 |

CVD-EVENTS

| A | B | P for Nonlinearity |
| --- | --- | --- |
| ALP | Partial adjustment | -/- |
|  | Fully adjustment | -/- |
| phosphate | Partial adjustment | 0.3387* |
|  | Fully adjustment | 0.1687 |

*4 knots spline estimation

P for Nonlinearity is not estimated to CVD deaths of ALP because all studies gave the RR per unit
